# Housing conditions and long-term care needs of older adults in Ghana: Evidence from the WHO SAGE Ghana Wave 1

**DOI:** 10.1371/journal.pgph.0000863

**Published:** 2022-12-07

**Authors:** Kofi Awuviry-Newton, Kwamina Abekah-Carter, Kwadwo Ofori-Dua, Razak M. Gyasi, Cindy Nhyira Newton, Williams Agyemang-Duah, Paul Kowal, Pablo Villalobos Dintrans

**Affiliations:** 1 African Health and Ageing Research Centre, Winneba, Ghana; 2 College of Health and Biomedical Science, Victoria University, Melbourne, Victoria, Australia; 3 Department of Social Work, University of Ghana, Legon, Accra, Ghana; 4 Department of Sociology and Social Work, Kwame Nkrumah University of Science and Technology, Kumasi, Ghana; 5 Aging and Development Unit, African Population and Health Research Center, Nairobi, Kenya; 6 Department of Public Health, Kwame Nkrumah University of Science and Technology, Kumasi, Ghana; 7 International Health Transitions, Canberra, Australia; 8 Facultad de Ciencias Médicas, Programa Centro Salud Pública, Universidad de Santiago, Santiago, Chile; Instituto Nacional de Geriatria, MEXICO

## Abstract

The present study examined the association between housing conditions and long-term care needs of older adults in Ghana. We used data from 4,920 adults aged ≥50 years that participated in the World Health Organisation’s (WHO) Study on adult health and AGEing Ghana Wave 1. Housing conditions were assessed with drinking water, sanitation, cooking conditions and building materials, and long-term care needs were based on WHO Disability Assessment Schedule 2.0. Multivariable logistic regressions modelled the effect of housing conditions on long-term care needs. After full adjustment for all available potential confounders, older adults living in households with unimproved cooking conditions had higher odds of reporting long-term care needs (OR = 6.87, 95%CI: 5.04–9.37) compared to those in improved cooking condition households. Moreover, those in households with unimproved housing materials (OR = 1.27, 95%CI: 1.01–1.72) and those in unimproved sanitation households (OR = 1.26, 95%CI: 1.05–1.54) were more likely to experience long-term care needs after respectively controlling for demographic and health-related covariates. Poor housing conditions are risk factors of long-term care needs in Ghana. Efforts to improve housing conditions may benefit older age functional abilities and unmet long-term care needs.

## Introduction

Robust geriatric assessment of the care needs of older adults requires an understanding of a person’s physical and mental health status and their context, including the housing conditions. The United Nations Decade of Healthy Ageing (2021–2030) aims to prolong older adults’ life expectancy with good health by addressing four areas of action [1]. The first three action areas are to alter the way we think, feel and act towards age and ageing; develop communities in ways that foster the abilities of older people; and deliver person-centred, integrated care and primary health services that are responsive to older people [1]. The fourth of the action points seeks to provide access to long-term care services for older adults who need it the most [1]. However, understanding the long-term care needs of older adults is relevant to providing long-term care services that can address their unique needs. The current study was inspired by Gu and Vlosky’s [2] study conducted in China, where disability in six activities of daily living (bathing, dressing, indoor transferring, toileting, incontinence, and eating) was used to measure long-term care needs of older adults. Following this understanding, the current study employed the World Health Organisation Disability Assessment Scale (2.0), which measured disability with 12 variables across six domains of cognition, mobility, self-care, getting along with others, life activities, and participation in society (S1 Appendix) to measure long-term care needs. The support older adults may require for more than six months due to a prolonged disability or frailty has increasingly become important among ageing populations globally [3]. Compared to the advanced countries where governments allocate budgets for the provision of long-term care for older persons [4,5], the primary provider of long-term care for older adults in Ghana has always been family members as the state is yet to give this the upmost priority [6,7]. Moreover, the few available private home care facilities for older persons in the country are not highly patronized as the cost of their services are considered expensive [4].

Conceptualized as the construction and assigned usage of the built environment or architecture and interior design facilities collectively including sanitation, water, floor type, for sheltering people and providing comfort, housing has been identified as a critical condition for achieving the long-term care needs of many older adults [8]. Several studies have evaluated the effects of congregate housing on health outcomes of the general population [9,10] and older residents in particular [8,11,12]. However, literature regarding the relationships of housing with long-term care needs of older adults in many low- and middle-income countries, including Ghana, are few.

Ghana’s population is growing older, with increased concerns for the long-term care needs of older adults [13,14]. Whiles the overall life expectancy (LE) for Ghanaians in 2019 was 63.9, a 0.41% increase from 2018 (63.7), healthy life expectancy (HLE) at birth was 58.0 years, 2.82% rise from 2016 (56.2) [15]. The gross percentage increase in HLE being higher than the overall percentage increase in LE signifies an improvement in the functional ability of older adults; however, a huge proportion of older adults will require long-term care from disability. The continuing life expectancy increase seen in Ghana has raised concerns about older adults’ long-term care needs [16]. Older adults in Ghana report higher levels of disability in general compared to other middle-income countries such as China and South Africa [17]. An investigation of the link between housing and long-term care needs among increasingly vulnerable older adults in a developing country context may be relevant for social policy and applied gerontology to ensure optimal well-being for older adults.

While evidence on the relationship between housing and health is well-established, comparatively little evidence exists about the relationship between housing and long-term care needs. In Ireland, a study reported that the value and suitability of older adults’ housing are essential to their living standards and can indicate whether or not it is suitable for when they are dependent [18]. Poor housing is reported to relate to needing high long-term care as older adults living in poor housing have a high prevalence of long-term chronic conditions [12]. In the USA, Carnemolla, Bridge [19] report(s) a relationship between the structure and layout of a house and independence of older adults. Although these findings from Western countries explicitly show an association between housing and health, they offer some clues for further research into housing and long-term care needs. In low- and middle-income countries, studies on the association between housing and long-term care needs, or health are limited. A systematic review on effects of the use of kerosene on papers from low- and middle-income countries revealed that kerosene use poses health risk to users including older adults [20]. In Ghana, a study reported that the use of unimproved water sources and unimproved sanitations are significantly associated with increased risk of depression, particularly among women.^37^ The current study extends the available evidence in low-and middle-income countries by exploring the association between housing conditions and long-term care needs of older adults in Ghana.

Regarding the confounding variables, available evidence shows that wealth (measured in income) [21,22], advanced aged [23,24], sex, education, multimorbidity, and marital status [24] influence older adults’ housing status and long-term care needs. Accordingly, this study examined the relationship between housing conditions and the long-term care needs of older adults, controlling for potential demographic and health-related factors. This study hypothesised that housing conditions will have an association with long-term care needs.

## Methods and material

### Study sampling

We used a nationally representative data from the Study on global AGEing and adult health (SAGE) Ghana Wave 1 conducted between 2007/2010 across all the 13 regions of Ghana (S1 Data). The sample size recorded in the study was 5,571 participants (aged 18 years and older); however, this study used a sample of 4,920 participants who responded to all 12 questions on functional disability, which served as a proxy for long-term care need for our analyses. Additional details about the study methodology and other relevant information can be found elsewhere [25]. In addition, we merged the household dataset with the individual dataset for inclusion of housing variables, including water supply source, sanitation, cooking conditions, and housing construction materials, used to define ‘improved housing’. Ethical approval for this study was obtained from the WHO Ethical Research Committee.

### Long-term care needs

Long-term care needs were defined by the 12-item version of WHO Disability Assessment Schedule (WHODAS) 2.0, originally measured using five ordinal scales (none, mild, moderate, severe, and extremely severe). Disability is considered the best proxy for measuring long-term care because evidence shows that disability necessitates older adults’ long-term care needs [26]. The WHODAS 2.0 contains 12 questions from all six domains of the full version, namely: cognition, mobility, self-care, getting along, life activities, and participation in society [27]. S1 Appendix contains the 12 questions included in the analysis. We scored the WHODAS 2.0 on a scale of 0 to 100, with a lower score implying lower disability and a higher score, high disability. Similar to other published studies [10–12] we decided to take the top 10^th^ percentile as our cut-off point for determining the severity of the disability. That is, scoring <90.18% were considered “no disability” and “> = 90.18%” were considered as “with a disability” [11,12]. In our study, we used the disability score to determine the long-term care needs of older adults. That is, those “with a disability” > = 90.18%” disability score) were considered as “needing long-term care”. Long-term care, in this case, will include assistance with personal care, household chores as a result of limitations experienced by individuals spanning from a longer period. Using this cut-off point is understandable as individuals reporting more than moderate to extremely severe on any of the functional activities usually require some form of care [15]. Individuals reporting “no disability” <90.18% disability score) were considered “not needing long-term care”. It is not unusual for researchers to use disability severity as a proxy for measuring long-term care needs. For instance, a report by WHO measured long-term care needs by considering the limitations on the five components of the WHO-ICF including body functions and structures, activity limitations as well as participation restrictions [1].

### Independent variables

#### Housing quality

Housing quality was measured using four variables, including questions about sanitation, water supply source, cooking conditions, and housing construction materials. As defined by the WHO Joint Monitoring Programme (WHO-JMP) [28], when all four variables met the criteria for improvement (detail given below), overall housing quality was considered as improved [29,30]. In this study and similar to Awuviry-Newton, Wales, Tavener, Kowal, Byles [14], we decided to measure the effects of the four separate variables on the long-term care needs of older adults, rather than using the overall measure of housing quality. This technique is relevant because it enlightens the unique impact and quantum of association on long-term care. Definitions of improved are provided for each of the individual components below.

- Sanitation
  In SAGE Ghana Wave 1, sanitation was measured through *the type of toilet facility* and *whether the toilet is shared* as categorised by the WHO-JMP [16]. The question *“what type of toilet facility do members of your household usually use*?” was used to determine *“toilet facility type”*. Based on the WHO-JMP categorisation, ‘improved sanitation’ included: 1) flush/pour-flush to a piped sewer system, flush/pour-flush to a septic tank, flush/pour flush to pit latrine, flush/pour flush to other location, flush/pour flush to unknown place/not sure, ventilated improved pit latrine, pit latrine with slab, and composting toilet. On the other hand, households with the following types of toilets were considered ‘unimproved sanitation’: pit latrine without slab/open pit, bucket latrine, hanging toilet/hanging latrine, no facilities or bush or field. The question, “Do you share this facility with other households?” was used to assess the *‘toilet shared’* expressed in dichotomous response (yes or no). When participants responded ‘no’, the variable was defined as ‘improved’ whereas ‘yes’ was defined as ‘unimproved’. Sanitation (improved vs unimproved) was measured by combining toilet shared (improved vs unimproved) and toilet facility type (improved vs unimproved). Combining these two variables, sanitation was considered as “improved” if the participant reported improved for toilet shared and toilet facility type. On the other hand, sanitation was considered unimproved if the participant reported unimproved in at least one of the two variables.
- Drinking water source
  To determine the water supply source, the question, *“What is the main source of drinking water for members of this household*?*”* was asked, with 13 possible response categories. “*Improved water supply*” included piped water into dwelling, piped water to yard/plot, public tap/standpipe, tube well/borehole, protected dug well, protected spring, hygienic source bottled water, and rainwater collection. *Unimproved water supply* included unprotected dug well, unprotected spring, small scale vendor, tanker-truck, surface water (river, dam, lake, pond, stream, canal, irrigation channels) [16].
- Cooking conditions
  Three variables were used to assess the quality of the cooking conditions in the household, including a question about *where cooking takes place*, *what the food was cooked on*, and *cooking fuel used to cook food*. Where cooking took place was measured with the response categories *“cooking in the living or sleeping room”*, *“outdoor”*, and *“in a separate room”*. Cooking in the living or sleeping room and outdoors was considered “unimproved”, and cooking in a separate room was considered *“improved”*. The second question was, “*In this household*, *is food cooked on an open fire*, *an open or closed stove*?*”*. Again, we considered *“open fire”* as “unimproved” and *“open stove”* and *“closed stove”* as “improved”. Lastly, the question *“What type of fuel does your household mainly use for cooking*?*”* “Unimproved” included coal/charcoal, wood, agriculture/crop, animal dung, shrubs/grass, while “improved” included gas, electricity, and kerosene/paraffin.
  Cooking conditions were therefore classified as “unimproved” when participants reported *unimproved* in at least any two of the three variables whereas “improved” referred to the opposite [16].
- Construction material quality
  Two questions were included about the *floor* and *wall type* to measure the *quality of construction materials*. *Floor type* was measured with the question “*What type of floor does your dwelling have*?’ categorised as *“hard floor”* (tile, cement, brick, wood) and *“earth floor”*. Hard floor” was considered as *“improved”* and “earth floor” as *“unimproved”*. For the wall type, responses to the question, “*What type of wall does your dwelling have*?” included 1) cement brick, stone, or wood; 2) mud/mud brick; 3) thatch and other; 4) plastic sheet; 5) metal sheet. Per the categorisation of WHO [16], we categorised them as *“unimproved”* (mud/mud brick; thatch and other; plastic sheet, metal sheet) and *“improved”* (cement, brick, stone, or wood). Construction material was considered as *“unimprove*d” if participants reported unimproved in at least one of the two variables”. Otherwise, we considered it as *“improved”* if we categorized both variables as improved.

#### Confounding adjustment

A set of socio-demographic and health-related variables was considered as confounding factors. The socio-demographic characteristics considered were the age of participants (in years), education (no education, maximum junior high completed, at least senior high achieved), marital status (single, separated, divorced, married, cohabiting, widowed), sex (male, female), location of residence (rural/urban) and relative wealth (measured in quintiles (Q1-Q5) [31]. Relative wealth was measured using household assets and possessions [31,32]. Quintile 1 refers to the household with the poorest states, whereas Quintile 5 referring to the richest household.

The health variables used as covariates in the analyses were self-reported conditions compiled into multi-morbidity status (no chronic conditions, single condition, more than two conditions). Chronic conditions included in this analysis were stroke, arthritis, angina, diabetes, chronic lung disease/asthma, hypertension, cataract, oral health, and injuries. Body mass index (BMI) was also categorised as underweight (<18.5 kg/m^2^), normal weight (18.5–24.9 kg/m^2^), overweight (25.0–29.9 kg/m^2^) and obese (⩾30.0 kg/m^2^).

#### Analysis

Descriptive analyses, including frequency and percentages, were used to describe the categorical variables, whereas mean and standard deviation were used for the continuous variable, particularly age. Chi-square and t-test were used to test the relationships between independent variables and the generated dependent variable (long-term care needs). Bivariate and multivariate logistic regressions were performed to estimate the crude and adjusted Odds Ratios (OR) and 95% confidence intervals (CI) for the associations between housing variables and long-term care needs. All variables that were at *p*<0.05 on the bivariate analyses were included in the multivariable logistics regression model. We conducted a multivariate logistic regression adjusting for all available potential confounders to examine whether the associations between housing variables and long-term care needs were independent of sociodemographic and health factors. We developed four models of logistic regression. Model 1 –unadjusted relation between housing quality variables and long-term care needs; Model 2 –adjusted for sociodemographic variables (age, sex, marital status, location of residence, education, relative wealth; Model 3 –adjusting for health variables; and Model 4 –adjusting for all socio-demographic and health related variables at a p-value<0.20. STATA 16 was used as a statistical software package for the analysis.

### Ethical consideration

Ethical approval for this analysis was obtained from the World Health Organization Ethical Research Committee. Written informed consent was obtained from all participants.

## Results

### Descriptive statistics

Bivariate analysis of independent variables in relation to long-term care needs is presented in Table 1. Most participants were men (53.7%), with a mean age of 60 years. More than half were married/cohabiting (60.4%), lived in a rural area (59.3%), and 48.2% had no formal education. Nearly the same proportion of participants was distributed across the income quintiles, with a little over one-quarter reporting the highest income quintile (20.6%). About 68% reported having no chronic conditions, and nearly half had a normal BMI (55.7%). Nearly 12% of participants reported needing long-term care, with prevalence relatively significant. Comparatively, very few of the participants were living in unimproved housing conditions: 17.6% lived in households with unimproved water source, 32.2% lived in households with an unimproved sanitation, and 13.5% with unimproved housing construction materials. Over 93% had unimproved cooking conditions (93.4%).

**Table 1:** Bivariate analysis of independent variables in relation to LTC need.

Independent variables	Overall	LTC need		*p*-value
	N(%)	No, N (%)	Yes, N (%)	
**Age (Mean, SD)**	59.8±14.1	58.4±13.5	70.5±13.9	**<0.001**
**Sex**				**<0.001**
Male	2642 (53.7)	2,413 (91.3)	229 (8.67)	
Female	2278 (46.3)	1,931 (84.8)	347 (15.2)	
**Marital status**				**<0.001**
Never married	140 (2.87)	123 (87.9)	17 (12.1)	
Married/cohabiting	2,946 (60.4)	2,737 (92.9)	209 (7.09)	
Separated/divorce	640 (13.1)	569 (88.9)	71 (11.1)	
Widowed	1152 (23.6)	902 (78.3)	250 (21.7)	
**Location of residence**				0.565
Rural	2918 (59.3)	2,570 (88.1)	348 (11.9)	
Urban	2002 (40.7)	1,774 (88.6)	228 (11.4)	
**Education**				**<0.001**
No education	2,357 (48.2)	1,955 (82.9)	402 (17.1)	
At most primary education completed	1,177 (24.1)	1,086 (92.3)	91 (7.73)	
Senior high completed	1,185 (24.2)	1,116 (94.2)	69 (5.82)	
University degree/post	172 (3.52)	162 (94.2)	10 (5.81)	
**Income quintiles**				**<0.01**
Q1 (lowest wealth)	955 (19.4)	831 (87.0)	124 (13.0)	
Q2	967 (19.7)	851 (88.0)	116 (12.0)	
Q3	973 (19.8)	835 (85.8)	138 (14.2)	
Q4	1,014 (20.6)	909 (89.6)	105 (10.4)	
Q5 (highest)	1,004 (20.4)	913 (90.9)	91 (9.06)	
**Health variables**				
**Multi-morbidity**				**<0.001**
No conditions	3351 (68.1)	3,049 (91.0)	302 (9.01)	
Any 1 condition	1091 (22.2)	939 (86.1)	152 (13.9)	
Any 2 or more conditions	478 (9.72)	356 (74.5)	122 (25.5)	
**BMI**				**<0.001**
Normal weight	2738 (55.7)	2,466 (90.1)	272 (9.93)	
Underweight	687 (14.0)	560 (81.5)	127 (18.5)	
Overweight	911 (18.5)	829 (91.0)	82 (9.0)	
Obese	580 (11.8)	485 (83.6)	95 (16.4)	
**Housing variables**				
**Water supply source**				0.846
Improved	4052 (82.4)	3,579 (88.3)	473 (11.7)	
Unimproved	895 (17.6)	762 (88.1)	103 (11.9)	
**Sanitation**				0.359
Improved	3337 (67.8)	2,956 (88.6)	381 (11.4)	
Unimproved	1583 (32.2)	1,388 (87.7)	411 (12.3)	
**Cooking conditions**				**<0.001**
Improved	295 (6.58)	185 (62.7)	110 (37.3)	
Unimproved	4186 (93.4)	3,756 (89.7)	430 (10.3)	
**Housing construction material**				0.346
Improved	4256 (86.5)	3,765 (88.5)	491 (11.5)	
Unimproved	664 (13.5)	579 (87.2)	85 (12.0)	
**Outcome variable**				
**Long-term care needs**				
No	4,344 (88.3)	-	-	-
Yes	576 (11.7)	-	-	

Health-related variables and all demographic variables except the location of residence were associated with long-term care needs. Older women reported high long-term care need (61.7%) compared to their older men counterparts. Demographic characteristics, such as being widowed (21.7%), having no formal education (17.1%), being in the middle-income quintile (59.7%), living with at least two chronic conditions (25.5%), and being underweight (18.5%) reported higher long-term care needs compared to their respective counterparts. A slight variation of prevalence of long-term care needs existing between households with unimproved water supply and households with an improved water supply source (11.9% vs 11.7%). Likewise, a relatively high prevalence (12.3%) of long-term care need was found among participants living in households with unimproved sanitation. A positive association was found between unimproved cooking conditions and long-term care needs. participants living in housing constructed with unimproved housing construction materials had a relatively high prevalence of long-term care need (12.0%). The prevalence of long-term care needs in relation to specific variables across the 12-items are relatively high (Table 2).

**Table 2:** Prevalence of disability (long-term care needs) across the 12-item of WHODAS 2.0.

Disability (Long-term care needs)	None N (%)	Mild N (%)	Moderate N (%)	Severe N (%)	Extreme/cannot do at all N(%)
Difficulty learning a new task, for example, learning how to get to a new place	2,367 (46.4)	1,377 (27.0)	1,049 (20.6)	269 (5.27)	41 (0.80)
Difficulty making new friendships or maintaining current friendships	3,381 (66.3)	845 (16.6)	585 (11.5)	218 (4.27)	71 (1.39)
Difficulty dealing with strangers	3426 (67.2)	758 (14.9)	620 (12.2)	234 (4.59)	59 (1.16)
Difficulty standing for long periods, such as 30 min	2,033 (40.0)	1.213 (23.8)	1024 (20.1)	587 (11.5)	231 (4.54)
Difficulty taking care of your household responsibilities	2,807 (55.7)	918 (18.2)	750 (14.9)	334 (6.63)	229 (4.55)
Difficulty joining community activities (for example, festivities, religious or other activities) in the same way as anyone else can?	2,812 (55.4)	1,177 (23.2)	772 (15.2)	209 (4.12)	103 (2.03)
Difficulty concentrating on doing something for 10 min?	3,177 (62.4)	1,211 (23.8)	555 (10.9)	111 (2.18)	35 (0.69)
Difficulty walking long distance, such as one kilometre?	2,203 (43.5)	1020 (20.1)	969 (19.1)	528 (10.4)	348 (6.87)
Difficulty bathing/washing your whole body?	4,211 (82.7)	537 (10.6)	247 (4.85)	65 (1.28)	32 (0.63)
Difficulty getting dressed	4,219 (82.8)	507 (9.95)	288 (5.65)	51 (1.00)	31 (0.61)
Difficulty performing your day to day work	2,742 (54.2)	894 (17.7)	1,015 (20.0)	271 (5.35)	142 (2.80)
In the last 30 days, how much have you been emotionally affected by your health condition(s)?	2,569 (50.5)	1,356 (26.6)	872 (17.1)	246 (4.83)	47 (0.92)

### Housing and long-term care needs

Table 3 shows the multiple logistic regression analysis assessing the association between housing variables and long-term care needs. Although in model 1, the relationship was not significant, in model 2, after adjusting for significant socio-demographic variables, the relationship between the unimproved water supply and long-term care need remained statistically insignificant (*p* = 0.672). Similarly, in terms of relationship, after adjusting for health variables including BMI and multi-morbidity in model 3, an insignificant relationship between unimproved housing construction materials and long-term care needs was noticed. In the parsimonious model 4, after adjusting for significant variables from models 3 and 4, there was a slight reduction (3%) in the likelihood of long-term care need (AOR; 1.10: CI: 0.94, 1.43), although this relationship was not significant (Table 3).

**Table 3:** Result of the multiple logistic regression analysis assessing the association between housing variables and LTC needs.

Outcome Variable	Model 1		Model 2		Model 3		Model 4	
	Unadjusted odds (95% CI)	*p*-value	Adjusted odds (95% CI)	*p*-value	Adjusted odds (95% CI)	*p*-value	Adjusted odds (95% CI)	*p*-value
**Water supply source**		0.846		0.672		0.273		0.492
Improved	1		1		1		1	
Unimproved	1.02 (0.82, 1.28)		1.05 (0.82, 1.36)		1.13 (0.90, 1.44))		1.10 (0.94, 1.43)	
**Sanitation**		0.359		0.194		**0.016**		0.071
Improved	1		1		1		1	
Unimproved	1.09 (0.91, 1.31)		1.15 (0.93, 1.42)		1.26 (1.05, 1.54)*		1.22 (0.98, 1.52)	
**Cooking conditions**		**<0.001**		**<0.001**		**<0.001**		**<0.001**
Improved	1		1		1		1	
Unimproved	5.19 (4.02, 6.71)***		6.59 (4.86, 8.94)***		5.59 (4.28, 7.29)***		6.87 (5.04, 9.37)***	
**Housing construction materials**		0.346	0.111	**0.021**		0.084		0.112
Improved	1		1		1		1	
Unimproved	1.23 (0.88, 1.44)		1.27 (1.01, 1.72)***		1.25 (0.97, 1.62)		1.29 (0.95, 1.75)	

Note: Model 1- Unadjusted model; Model 2 –Adjusted for Socio-demographic variables; Model 3 –Adjusted for health variables; Model 4 –Adjusted for Socio-demographics and health variables.

*** p<0.001;

**p<0.01;

*p<0.05.

Participants living in households with the unimproved sanitation were 9% more likely to have long-term care needs in model 1. After controlling for socio-demographic variables, the relationship remained statistically insignificant (AOR, 1.15; CI: 0.93, 1.42). Adjusting for health variables in model 3, participants living with unimproved sanitation were 26% more likely to have long-term care need with a statistically significant relationship (*p*<0.01). When adjusted for all significant cofounding variables, participants living in households with us were 22% more likely to have long-term care need compared to their counterparts (*p* = 0.07).

Participants in households with unimproved cooking conditions were 419% more likely to report long-term care need than those living in households with improved cooking conditions (p<0.001) in model 1. When adjusted for socio-demographic variables in model 2, the relationship remained significant at p<0.001 (AOR, 6.59; CI: 4.86, 8.94). When adjusted for health variables in model 3, participants in households with unimproved cooking conditions were 459% more likely to have long-term care need compared to that reported improvement (p<0.001). In model 4, when adjusted for all significant socio-demographic and health variables, a significant independent relationship was maintained between unimproved cooking conditions and long-term care need (AOR; 6.87, CI: 5.04, 9.37).

Participants living in households with unimproved housing construction materials were 23% more likely to have long-term care need (p = 0.346) in model 1. When we adjusted for the significant socio-demographic characteristics in model 2, participants living in households with unimproved housing construction materials were 27% more likely to have long-term care need than those living in housing with improved construction materials (p<0.05). However, the relationship became statistically insignificant when adjusted for health variables (AOR; 1.25: CI; 0.97, 1.62). This insignificant relationship persisted when adjusted for all significant variables in model 4 (AOR, 1.23; CI: 0.95, 1.75).

## Discussion

This study examined the relationship between housing conditions and long-term care needs in Ghana using data from the Study on global AGEing and adult health (SAGE) Wave 1. The results provide initial information about the relationship between housing and health and point to specific housing areas to address and subsequently be used to moderate the long-term care needs of older adults in the country. The current study has begun a debate on an innovative area of research by focussing on the relationships between housing and long-term care needs. The prevalence of long-term care needs found in this current study was nearly 12%. The prevalence seems low in this current study but it is likely to be high as the Ghanaian population grows. Overall, housing conditions except water supply source were associated with long-term care needs in models that adjusted for health variables but not for socio-demographic variables. This finding implies that health factors better moderate the relationship between housing and long-term care needs. Health-related factors determining the significant association between housing and long-term care needs, as revealed in this study, are not surprising because older adults living with multiple chronic conditions will have more functional disabilities [14,33]. that may increase their needs for long-term care. While water is considered a vital basic necessity, the plausible explanation for its non-significant association with LTC needs could be that it may be the least prioritized housing condition for the sampling units.

Sanitation was associated with long-term care needs when adjusted for health variables as opposed to demographic variables depicting that health-related factors may offer a better explanation on the relationship between housing (sanitation) and long-term care needs among older adults in Ghana. The finding that multi-morbidity and underweight led to a significant relationship between housing and long-term care needs increase our understanding of how the relationship may be health-related. This finding further implies that older adults living in poor sanitary conditions have a high need for long-term care as it harbours rapid transmission of diseases [34]. Evidence in Ghana shows that an appreciable number of older adults go to the toilet in the bush or fields, and some using open defecation around river bodies or bagged it in polythene during the day and throw it around the vicinity during the night [14,35]. The health of older adults is at risk should they remain in bad sanitary environments, which may increase the need for long-term care. Mandated state institutions should champion sanitation activities nationwide. Efforts geared towards ensuring that every household has an appropriate toilet and bath facility, as well as a proper waste disposal system, should be intensified as this may improve older adults’ long-term care experiences.

We found evidence that unimproved cooking conditions associate with increased long-term care needs of older adults in Ghana, affirming the current evidence of the harmful impact of cooking in the living room, using charcoal and wood for cooking on older adults’ health [36]. Cooking in household spaces like the living room instead of a kitchen, could expose older adults to serious air pollution and suffer respiratory complications [37,38]. It is significant to encourage the use of specified kitchens and modernized cooking apparatus like a gas stove in a household with older adults. A social services initiative specialised to ensure the affordability of closed stoves could increase the patronage, thereby reducing their long-term care need. A specialised strategy by stakeholders including governmental and non-governmental organisations to improve the cooking conditions of households with older adults can contribute to meeting older adults’ long-term care needs in Ghana. A long-term care policy that can specifically provide access to caregivers of older adults with modernised cooking stoves either free or at reduced cost will help promote enhancing their ageing experience.

Housing material was associated with long-term care need when adjusted for socio-demographic factors implying that socio-demographic factors such as advanced age and income affect the relationship. The quality of the housing environment, the physical nature of the housing, and the presence of vital amenities are relevant factors to be considered in assessing a household’s housing condition [39]. Nonetheless, building materials in Ghana are generally expensive relative to the income of many older adults [40,41], compelling many to purchase poor building materials that are likely to deteriorate faster. Additionally, when poor building materials are used in constructing a house, it could pose a threat to the safety of older adults. For instance, houses built with mud could have damp conditions, which may cause cracks in the wall [37]. Therefore, it would be useful if older adults are assisted in having access to proper building materials that would enable them to construct elderly-friendly houses to make their living comfortable. A voluntary amount of money can be contributed towards older adults’ savings (irrespective of their employment status) by the state to cater to their housing needs, especially during the later years of life.

To the best of our knowledge, this study is the first to use a countrywide sample to examine the relationship between housing conditions and long-term care needs among older adults in Africa and developing countries. The current study has begun a new area of research interest into the association between housing and long-term care needs. The present study raises a new question that requires further examination. Specifically, how do the association of housing condition and long-term care needs differ according to gender, age, and chronic conditions? On a positive note, the current study provides baseline information towards the improvement of housing conditions that may enhance long-term care experiences among older adults.

### Limitations of this study

Some limitations of this study need to be acknowledged. The first limitation is that we used functional disability to measure long-term care needs, which could have been measured from self-reported health, which elucidates our understanding of how older adults yearn for long-term care. Second, the approach we adopted to categorise responses for long-term care may be misclassified; however, we ensured to also classify those reporting needing no care and those needing mild care. Although the current study suggests demographic and health impacts of the association between housing conditions and long-term care needs, it did not examine how gender and chronic conditions influence the association. Moreover, the research did not capture the causal effect of the association between housing and long-term care needs.

## Conclusions

The current study provides a baseline finding for older adults’ housing and long-term care needs in this decade of healthy ageing. This research provides a basis for policymakers to focus attention on practical housing policies and programmes to ensure improved housing conditions to safeguard the health and well-being of older adults. Further studies on housing needs of older adults to their long-term care may benefit from longitudinal analysis and qualitative data to inform policymakers’ understanding of the need to care for older adults.

## Supporting information

S1 Appendix List of the 12 variables included in the WHODAS 2.0 score. (DOCX)

S1 Data Minimal dataset. (XLSX)

S1 Text Inclusivity in global research. (DOCX)
