# Supplementary material for: Housing conditions and long-term care needs of older adults in Ghana: Evidence from the WHO SAGE Ghana Wave 1
Source: PLOS Glob Public Health. 2022 Dec 7;2(12):e0000863. doi: 10.1371/journal.pgph.0000863 (PMC10021768; doi:10.1371/journal.pgph.0000863)
Supplement: S1 Appendix — (DOCX) [file pgph.0000863.s001.docx]

**Appendix A. List of the 12 variables included in the WHODAS 2.0 score**

| **In the last 30 days how much difficulty do you have in:** | |
| --- | --- |
| **…learning a new task, for example, learning how to get to a new place?** | None = **0**, Mild = **1**, Moderate = **2**, Severe = **3**, Extreme/cannot = **4** |
| **…making new friendships or maintaining current friendships?** |  |
| **…dealing with strangers?** |  |
| **…standing for long periods, such as 30 min?** |  |
| **…taking care of your household responsibilities?** |  |
| **…joining community activities (for example, festivities, religious or other activities) in the same way as anyone else can?** |  |
| **…concentrating on doing something for 10 min?** |  |
| **…walking long distance, such as one kilometre?** |  |
| **…bathing/washing your whole body?** |  |
| **…getting dressed?** |  |
| **…performing your day to day work?** |  |
| **In the last 30 days, how much have you been emotionally affected by your health condition(s)?** |  |
